# Recent advances in understanding/managing type 2 diabetes mellitus

**DOI:** 10.12688/f1000research.11192.1

**Published:** 2017-10-31

**Authors:** Pablo Aschner

**Affiliations:** 1 Javeriana University and San Ignacio University Hospital, Bogota, Colombia

**Keywords:** type 2 diabetes mellitus, mechanisms of drug action, glucose-lowering drugs

## Abstract

The treatment of type 2 diabetes mellitus has evolved in the present century toward safer and maybe more effective drugs, which in some cases can also reduce the risk of cardiovascular and renal outcomes. Nevertheless, we still need better strategies to reduce excess body weight in order to achieve diabetes remission, which is now a feasible target, as has been demonstrated with bariatric surgery. This review focuses on the significant advances in the management of blood glucose in type 2 diabetes mellitus, including the current understanding of the mechanisms of drug action but keeping in mind that the treatment of the disease is multifactorial.

Until the middle of the last century, there was little distinction in the treatment of diabetes mellitus among people with different ages or phenotypic characteristics because only insulin was available. Although it was recognized that pancreatic failure was not the only cause, there was no other sufficient explanation ^1^. The roles of heredity and obesity were known and there were attempts to tackle the latter by changes in diet. At that time, the first-generation sulfonylureas (SUs) were developed as a successful oral replacement of insulin in most people with what we now know as type 2 diabetes mellitus (T2D). Ten years later, a study run by a university group (comparing tolbutamide with insulin or diet alone) suggested that SUs might increase cardiovascular (CV) mortality but was harshly criticized ^2^ and eventually disregarded with the arrival of second-generation SUs (glibenclamide/glyburide, glipizide, gliclazide, and glimepiride), which are still widely used. By the end of the last century, the UK Prospective Diabetes Study (UKPDS) ^3^ demonstrated their CV safety (using chlorpropamide and glibenclamide/glyburide) but their main caveats have been the risk of hypoglycemia (particularly with glibenclamide/glyburide), increased weight, and maybe shorter duration of the effect in the long term when compared with other glucose-lowering drugs (GLDs) ^4^. The attempt to minimize hypoglycemia by developing short-acting secretagogues such as the glinides has not been very successful.

By the same time that SUs started to be used, the first biguanide (phenformin) was also launched but was withdrawn in most markets in the late ‘70s because of fatal cases of lactic acidosis. On the other hand, metformin, which was discovered even earlier and is much safer, has been increasingly used and now most guidelines consider it the preferred first-line GLD and the best partner in combination therapy. Results from the UKPDS suggested a pleiotropic effect, since the metformin arm was the only one in which the incidences of mortality and myocardial infarction were reduced in the first 10 years when given to overweight people, even though the mean difference in HbA1c against conventional treatment was lower than in the main trial with SUs or insulin ^5^. It was also the only one which did not increase weight, and it is on the World Health Organization’s list of essential medicines. Its mechanisms of action (MOAs) are still being discussed, but it has been considered essentially an insulin sensitizer (at least in the liver).

Insulin resistance gradually took over the “pancreatocentric” approach as a target for the treatment of T2D, particularly at the beginning of this century when thiazolidinediones (TZDs) were introduced as the first true insulin sensitizers. They are peroxisome proliferator-activated receptor (PPAR) gamma receptor agonists predominantly in the adipose tissue, and their main MOA is reducing lipotoxicity by “stealing” free fatty acids and increasing adiponectin ^6^. But they also have anti-inflammatory effects that could be anti-atherogenic. Their CV safety has been under scrutiny, and in fact rosiglitazone had to be withdrawn under the suspicion of CV harm ^7^, which since has been refuted, especially by one randomized clinical study ^8^. The other, pioglitazone, has shown CV benefit by reducing major adverse CV events (MACE) as a main secondary endpoint in patients with CV disease ^9^ and by reducing stroke or myocardial infarction in patients with insulin resistance and a history of ischemic stroke or transient ischemic attacks ^10^, but the number needed to harm (mainly heart failure, edema, and serious fractures) may be unacceptable. Pioglitazone is still being used in a minority of patients and its main benefit remains probably in non-alcoholic steatohepatitis ^11^. TZDs also increase weight more than other GLDs but may change the body fat composition in favor of less visceral fat ^12^. Dual TZDs acting on PPAR gamma and alpha receptors (glitazars) could add a beneficial effect on lipids, but the clinical results have been conflicting and only a few remain in exploratory phases ^13^. Pan PPAR receptor agonists (gamma, alpha, and delta) have been studied considering that the increased fat oxidation promoted by PPAR delta might avoid increased adiposity and weight gain, but their safety is an issue and so far these agonists serve only as a proof of concept ^14^.

Given the complicated story of TZDs, metformin remains the only widely used GLD to treat insulin resistance. But in fact it is an insulin sensitizer only at the level of liver-reducing endogenous glucose production in comparison with TZDs, which act at both levels (liver and periphery) ^15^. Recent studies with a delayed-release preparation of metformin have shown that although there is considerably less absorption of the drug at the level of the ileum, the efficacy is similar to the extended-release preparation, suggesting that its main action could be intraluminal by stimulating the production of glucagon-like peptide (GLP-1) and peptide YY at the level of the ileum (where the concentration of L-cells is high) ^16^. This preparation of metformin might prove useful in renal failure, which is now a contraindication for the use of this drug.

We are still left with the issue of how to reduce insulin resistance, and the only effective strategy is to reduce sufficient weight to abolish the excess of visceral fat. In fact, bariatric surgery has served as a proof of concept, since the weight loss is considerable and leads to a remission rate of up to 70% but varies depending on the type of surgery and the center ^17^. Complete remission defined by an HbA1c of less than 6% without GLD during 6 months should now be the ultimate target of diabetes mellitus control, and besides surgery there is little progress in this field. Unfortunately, surgery is not free of adverse events, and although they can be minimized by reaching the top of the learning curve and maintaining it in each surgical group, there are still complications such as stenosis, gastric fistula, gastroesophageal reflux, ulcers, intestinal obstruction, and nutritional and vitamin deficiencies.

There is an urgent need to develop anti-obesity drugs (or devices) that approach the results of bariatric surgery. Meanwhile, surgery, particularly when done early, is being recommended by some guidelines in patients with T2D and with a body mass index of more than 30 kg/m ^2^ ^18, 19^. Weight regain after any weight-losing strategy is also an unresolved problem ^20^. Up to one-third of bariatric surgery patients regain weight, and in one study with intensive lifestyle intervention where partial or complete remission could be observed initially in almost 12% of the subjects, there was considerable weight regain after the first year and this was probably the reason why the study failed to demonstrate reduction of CV outcomes ^21^.

Although improvement of beta cell function has been elusive, enhancement of insulin production in a glucose-dependent manner is now possible with the incretin effect mediated mainly by GLP-1 (and also by glucose-dependent insulinotropic polypeptide, or GIP). The main effect of GLP-1 is to enhance insulin secretion, but it ceases when blood glucose levels reach the normal range, thus avoiding hypoglycemia. The half-life of native circulating GLP-1 is extremely short because it is degraded by the enzyme dipeptidyl-peptidase-4 (DPP-4) but can be delayed by inhibition of the enzyme (DPP-4 inhibitors) or by developing GLP-1 receptor agonists resistant to the enzyme ^22^. For their full effect, DPP-4 inhibitors must inhibit 70 to 90% of the enzyme and this can be sustained for 24 hours with most drugs of this class. Incretin action of the GLP-1 receptor agonists can last for up to 1 week. DPP-4 inhibitors (when combined with metformin) increase blood levels of GLP-1 by twofold or threefold, and since their efficacy is similar to SUs without causing hypoglycemia or weight gain, they are becoming the preferred oral insulin secretagogues in most guidelines ^23^. GLP-1 receptor agonists increase GLP-1 actions up to 10-fold and, at this supraphysiological level, also reduce appetite and delay gastric emptying. Clinical trials show significant weight loss, but the main caveats are cost of injection and gastric side effects (nausea and vomiting). GLP-1 receptor agonists have been divided into short-acting (exenatide and lixisenatide) and long-acting (exenatide long-acting release, liraglutide, albiglutide, dulaglutide, and semaglutide) ^24^. The former produce better post-prandial glucose control but less weight loss. Both short-acting (lixisenatide) and long-acting (liraglutide) GLP-1 receptor agonists are now available as “fixed-ratio” combinations with basal insulin (glargine and degludec, respectively), and the main benefits are less risk of hypoglycemia and blunting of insulin-induced weight gain ^25^. Some of these peptides are being studied for nasal or oral administration.

Both classes of incretin-mediated GLD have been shown to be safe in long-term studies designed to meet the regulatory agencies’ requirement to prove CV safety, although these GLDs should not be used in patients with a history of chronic pancreatitis because there is still controversy on this issue. Recently, two CV safety trials—with liraglutide ^26^ and semaglutide ^27^—went beyond proving non-inferiority to demonstrate superiority by reducing CV events. Most of the effect can be considered pleiotropic, although there was a significant difference in HbA1c and weight between the active and the placebo group, particularly with the highest dose of semaglutide. There was also an increased incidence of retinopathy complications with the latter, and this may be due to the fast drop in blood glucose levels which may affect the retinal blood supply.

The inhibitory effect of GLP-1 on glucagon may be almost as important as the incretin effect on insulin and has raised the importance of that underestimated hormone in the physiopathology of diabetes mellitus ^28^. That may explain why some trials have shown benefit in patients with type 1 diabetes mellitus and glucagon is now a target for new GLDs. Drugs that block the glucagon receptor may lower blood glucose but most are only in the experimental phase ^29^. On the other hand, hybrid peptides acting on GLP-1 and glucagon receptors such as oxyntomodulin may enhance weight loss and, if the right balance can be found, might not affect the benefit of GLP-1 on glucose control ^30^ or even improve it by adding other peptides such as GIP ^31^.

The last class of drugs being introduced for the treatment of diabetes mellitus are the sodium-glucose co-transporter 2 (SGLT2) inhibitors, which inhibit glucose and sodium reabsorption in the proximal part of the kidney tubule. Up to 60 g/day of glucose will be excreted, leading to an insulin-independent decrease in blood glucose levels and loss of calories and thereby to weight reduction. The inhibition of SGLT2 is not complete (around 50%) and is partially compensated for by an increase in the activity of SGLT1 further down the tubules ^32^. Inhibitors of SGLT2 and SGLT1 are being developed, but their balance is still an issue. There is also an increase in endogenous glucose production, probably due to glucagon stimulation which can be avoided by combining them with incretin-mediated GLDs. The SGLT2 inhibitors also decrease blood pressure, but the mechanism is not clear. It may be due to the natriuretic effect, although it is short lived, or to osmotic diuresis or other MOAs ^33^. A recent CV safety study with empagliflozin went beyond proving non-inferiority to demonstrate superiority against placebo by reducing a composite of CV events (MACE) on top of the best current treatment in patients with CV disease. The effect can be considered pleiotropic and it started very early, suggesting a hemodynamic MOA which particularly decreased the rates of hospitalization for heart failure and mortality (secondary outcomes) ^34^. When added to standard care, it was also associated with slower progression of kidney disease and lower rates of clinically relevant renal events than was placebo ^35^. These findings were confirmed by the results of the CV safety trial with canagliflozin, which also showed a reduction of MACE and progression of renal outcomes ^36^.

There is still no full explanation for these results, and beyond the hemodynamic effect it has been hypothesized that by shunting substantial amounts of carbohydrate into the urine, there is a progressive shift in fuel utilization toward fatty substrates. The lower insulin-to-glucagon ratio favors glucose release and ketogenesis, and apparently ketones are preferred substrates for the heart and the kidney ^37^. But ketogenesis also may be a caveat since cases of normo-glycemic ketoacidosis have been reported, particularly in insulinopenic patients under stress. SGLT2 inhibitors increase the incidence of genital mycotic infections and may not be recommended when these infections are recurrent. Their glucosuric effect will become insignificant when glomerular filtration rate is low, and they are not recommended in stages 3B, 4, and 5 of renal failure. Significantly higher rates of spontaneous fractures and amputations were found in the canagliflozin study ^36^, and the MOA that leads to these infrequent but serious adverse events and whether it is a class effect is not yet clear.

In conclusion, there have been significant advances in the treatment of T2D which sometimes outpace our understanding of their MOA. On the other hand, we must keep in mind that the treatment of T2D is multifactorial, as demonstrated by the Steno 2 study, one of the best clinical trials to show a very significant reduction of mortality in T2D even beyond the initial intensive intervention (and, at the time it was done, none of the new GLDs was available!) ^38^.
